# Ecological History Shapes Transcriptome Variation in Quiescent *Saccharomyces cerevisiae*

**DOI:** 10.3390/biom15111588

**Published:** 2025-11-12

**Authors:** Agnieszka Marek, Katarzyna Tomala, Dominika Wloch-Salamon

**Affiliations:** 1 Institute of Botany, Faculty of Biology, Jagiellonian University, 30-387 Kraków, Poland; agnieszka.marek@uj.edu.pl; 2 Institute of Environmental Sciences, Faculty of Biology, Jagiellonian University, 30-387 Kraków, Poland; katarzyna.tomala@uj.edu.pl

**Keywords:** RNA-seq, quiescent cells, life history

## Abstract

Quiescence is a pivotal state for all living organisms and cells. However, recent research indicates a lack of uniformity among quiescent cells. That is, even if the primary feature of quiescence—the ability to restart divisions—is maintained, quiescent cells within populations exhibit variation in their cellular architecture and characteristics. While it is known that the process of entry into quiescence is influenced by a combination of nutrient starvation and temporal factors, the underlying mechanisms remain to be fully elucidated. In this study, we compare the transcriptomes of known homogenous fractions of dense quiescent yeast isolated from populations of different ecological histories. These populations have undergone experimental enrichment of certain types of quiescent cells during cycles of growth and starvation for 300 generations. Transcriptome analysis revealed discrepancies in terms of characteristics associated mainly with energy turnover processes, biosynthesis, and cell wall maintenance. The results of this study suggest that quiescent cells possess the capacity to adapt their transcriptome activity in accordance with their evolutionary history.

## 1. Introduction

Quiescence is a conserved form of the G0 state across various types of eukaryotic cells. Its hallmark is the cell’s ability to reenter the cell cycle after prolonged arrest. Understanding this omnipresent process of how cells enter, maintain, and exit quiescence and dormancy has significant implications not only for cancer biology and aging but also for all microorganism survival [1,2].

In single-celled organisms, quiescence could serve as an adaptive strategy enabling populations to withstand periods of starvation, regulate population heterogeneity, and engage in cooperative behaviors like resource recycling. Quiescence has fundamentally shaped microbial ecology by providing a mechanism for cells to predict and prepare for environmental changes, coordinate population responses through chemical signaling, and maintain community structure during periods of resource limitation, thereby serving as a cornerstone of microbial adaptation and survival strategies [3].

The biology of quiescent cells has important medical significance, notably in understanding human aging, stem cell function, and the pathogenesis of diseases, including cancer. In humans, the alternation between quiescence and proliferation not only governs tissue renewal, immune responses, and stem cell maintenance but also impacts tumor biology and persistent infections [2,4,5]. Quiescent cancer cells can evade anti-proliferative treatments and remain dormant in tissue niches, only to reactivate and drive metastasis—a major challenge in oncology. Moreover, quiescent microbial cells exhibit increased resistance to antibiotics and antifungals, complicating the treatment of chronic infections. Thus, elucidating the mechanisms and ecological context of quiescent cell states advances both fundamental biology and the development of more effective therapeutic treatments.

Recent research highlights that quiescence is not a static shutdown but rather a dynamic process marked by changes in cellular structure, metabolism, and phenotypic diversity, all of which contribute to evolutionary adaptation and population fitness. Quiescent cells exhibit distinct physiological and molecular characteristics, including reduced transcriptional activity, altered chromatin structure, and enhanced stress resistance, compared to growing cells [6]. The transition into and out of quiescence is tightly regulated by complex signaling networks, including the target of rapamycin (TOR), retinoblastoma (Rb), and cyclin-dependent kinase (CDK) pathways, which control cell cycle progression and metabolic adaptations in response to nutrient availability and stress signals [2,7]. Quiescence is also characterized by specific metabolic reprogramming, such as increased reliance on mitochondrial respiration and autophagy, which helps maintain cellular homeostasis during periods of inactivity [8]. Understanding these regulatory mechanisms is essential for biomedical applications, as dysregulation of quiescence has been implicated in pathological conditions, including cancer, where dormant tumor cells can evade chemotherapy and later contribute to relapse [7,9].

Although studied in various cells and organisms, budding yeast has emerged as a powerful model for studying quiescence in eukaryotic cells due to two key reasons: an experimental toolbox that makes it easy to isolate huge numbers of Q cells and study genes involved in quiescence regulation [10], and evolutionary conservation of many signaling pathways that regulate quiescence in yeast (e.g., TOR, PKA, and AMPK pathways) in higher eukaryotes, including humans [6]. Yeast cells transition into quiescence upon nutrient depletion (e.g., glucose or nitrogen starvation), and exit from the quiescence state is highly reproducible and easy to control in laboratory settings [11]. Yeast quiescent cells exhibit longevity and stress resistance, which provides insights into aging and dormancy mechanisms [12,13]. However, as in the other eukaryotes, yeast quiescence is not a uniform state—cells within a population exhibit different quiescent states, providing a model to study cellular heterogeneity and differentiation in quiescent populations. Observed heterogeneity at the level of populations and individual cells can result from the media used or the length of time in starvation [14,15].

There are currently no unified methods or protocols for studying observed quiescent heterogeneity [16]. However, the Werner–Washburne laboratory has demonstrated that, upon carbon exhaustion, a stationary-phase, haploid, planktonic yeast population can be separated into two subfractions: upper (U) and lower (L). Using a Percoll density gradient [11], these fractions exhibit distinct buoyant densities (approximate U = 1.10 g/mL and L = 1.14 g/mL), revealing clear morphological and physiological differences. The denser L fraction isolated by this method is homogeneous, mostly composed of smaller cells that show synchronous cell cycle reentry, and has high thermotolerance, enhanced viability over extended periods (>87% viability over 28 days), and a high reproductive capacity. The method allows for comprehensive transcriptomic and proteomic analyses, revealing differentially expressed mRNAs between L and the less-dense U fraction [17]. While we are aware that the fractionation method dichotomy fails to capture the whole complex of quiescent cell diversity that “displays various properties, shedding light on a plethora of individual behavior” [15], the dense fraction remains a valuable tool for comparative population-level studies [16].

We previously employed a fractionation method to elucidate the adaptive significance of phenotypic heterogeneity in starved *Saccharomyces cerevisiae* populations. To this end, we compared the survival and regrowth dynamics of monocultures composed exclusively of L or U quiescent cells versus naturally occurring mixed (U/L) quiescent populations across variable ecological conditions, including starvation duration and environmental complexity. L quiescent cells exhibit hallmark features of deep quiescence, accumulate high levels of storage carbohydrates (e.g., trehalose), display thickened cell walls and enlarged vacuoles, and maintain >80% viability after prolonged starvation (>4 weeks). U fraction cells show rapid loss of viability under the same conditions. The experimental data and mathematical modeling both confirm that L monocultures exhibit superior survival and shorter lag phases after long-term starvation, whereas U monocultures outperform L and mixed cultures following very short starvation and nutrient-recycling conditions [14].

Standard laboratory populations of S288c strains maintain rather stable proportions of U/L quiescent cells (15–20%/75–80%). However, when populations are experimentally enriched for one fraction by serially selecting only the U or L fraction over successive feast–famine cycles, the U/L balance evolves: in L selected lines progressively increase the denser fraction, while in U selected lines accumulate lighter cells, demonstrating that the U/L ratio is a heritable, evolvable trait shaped by ecological context [18]. Therefore, the evidence supports the hypothesis for U and L coexistence as a bet-hedging strategy that maximizes population fitness under fluctuating environmental pressures.

Several studies profiling the transcriptome of quiescent yeast cells as whole starved populations, as well as fractionated U and L fractions, confirm the presence of quiescent-specific expression patterns [19,20], global transcriptional shutdown, and the emergence of over 1000 noncoding RNAs specifically enriched in quiescence [21]. This finding indicates the potential existence of a distinct functional transcriptome “core program” involving quiescence. Additionally, if such a “core program” is present, it is expected to be manifested by a uniform L fraction. To date, no studies have directly compared the L quiescent fraction across populations that experienced distinct evolutionary histories during starvation conditions. This has led to the open question of whether and how the quiescent transcriptome adapts to varying environmental contexts.

In this study, we focused on a homogenous fraction of quiescent cells within lines differing by about 300 generations of varying selection-enrichment experimental procedures derived from our previous study [18]. The absence of discernible variation in the transcriptomics of these quiescent cells would support the notion of a “core quiescent” functional program maintained in the quiescence state. Nonetheless, the findings of this study suggest a more dynamic model of quiescence that exhibits a higher degree of adaptability to environmental stresses experienced.

## 2. Materials and Methods

### 2.1. Experimental Strains

This study utilized *Saccharomyces cerevisiae* strains obtained and characterized in our previous studies [18,22]. In short, the ancestral laboratory haploid strain S288c (*MATα*, *ura3::KanMX4*) [23] was used to establish six experimental lines. These populations underwent 300 generations of selection for enrichment for only one of two specific fractions of quiescent cells: U or L. The experimental regime consisted of alternating environmental conditions. During the *feast phase*, the cells grew on the rich nutrient plate as a colony, with free access to nutrients. The *starvation phase* began when the nutrients were gradually depleted, leading to a transition to quiescence due to nutrient deprivation. After 3.5 days, when the stationary phase was reached, populations were fractionated using a Percoll density gradient [11]. U and L fractions were separated, and one cell type was subjected to subsequent cycles of growth and starvation of Lines_U and Lines_L, respectively. From the final populations, we collected clones that differ in the proportions of U or L cells. For this study, we selected samples from this collection and grouped them based on the enriched quiescent cell type. The QU_group consisted of five clones. Clones were enriched for U cell proportions: U_3I = 50.24%, U_3III = 74.19%, U_4III = 53.01%, U_8III = 51.23%, and U_10III = 49.80%. The average proportion of U in this group of strains was UAv = 55.70% ± 9.31%. The QL_group included three clones enriched for L cells: L_2I = 91.27%, L_5I = 77.64%, and L_3II = 57.67%, with an average L cell proportion of LAv = 75.53% ± 16.90%. The average U fraction proportion from the ancestral population was 14% ± 3.3%. Whole-genome sequencing of these clones was performed in prior studies [18] and showed some SNVs acquired during the experiment (Supplementary Table S1). Genomic data were deposited in the SRA (NCBI BioProject: PRJNA383491, accession number SRP104610). Possible roles of the mutations are discussed in our previous papers [18,22].

### 2.2. Fractionation Assay

Selected clones were incubated overnight in rich medium at 30 °C with shaking. The following day, 20 μL of liquid culture (of each clone) was transferred to 20 mL of YPD medium and incubated with shaking at 30 °C for 7 days. Yeast cells were then separated into U and L fractions using Percoll centrifugation (as described previously [18]), and only the L fraction of quiescent cells was used. Post-centrifugation, quiescent L cells were collected from the bottom of the tubes and subjected to two sequential washes with 8 mL of Tris buffer. After each wash, the supernatant was carefully removed.

### 2.3. RNA Extraction and Purification from Quiescent Cells

The optical density (OD) of each sample was measured to ensure an extraction efficiency yielding approximately ≥20 ng/µL, corresponding to ~3 × 10^8^ cells. Total RNA was extracted from the isolated Q cells using the RiboPure™-Yeast Kit (Thermo Fisher Scientific, Waltham, MA, USA) following the manufacturer’s protocol. Each sample had two technical repetitions (1/2) that were then stored at −70 °C.

### 2.4. Transcriptome Analysis

Library construction, sequencing, and the subsequent bioinformatic analysis were carried out by Novogene. Lists of differentially expressed genes (DEGs) were created using the DESeq2 R package (version 1.36.0) [24]. They were then used to perform Gene Ontology (GO) enrichment analysis with the GOrilla tool (accessed on February 2025) [25]. The differentially expressed gene sets (with FDR < 0.05 and positive and negative fold change) were compared with background lists of all ORFs from the RNA-seq analysis. GO terms with padj < 0.05 in the Cellular Component, Molecular Function, and Biological Process categories were combined, and the mean values from two replicates were used to create heat maps. Gene expression heatmaps were generated based on FPKM values. For visualization, expression was scaled per gene by computing row-wise z-scores (mean = 0, SD = 1). Genes were grouped into predefined functional categories GO categories. Heatmaps were generated using the pheatmap package (version 1.0.13) in R, with clustering disabled. [26]. Heat maps were scaled by rows using the average linkage clustering method and the Pearson distance measurement method. Transcriptomic data were deposited in the GEO (Gene Expression Omnibus) Series GSE309454.

## 3. Results

### 3.1. Data Quality

**Figure 1:**
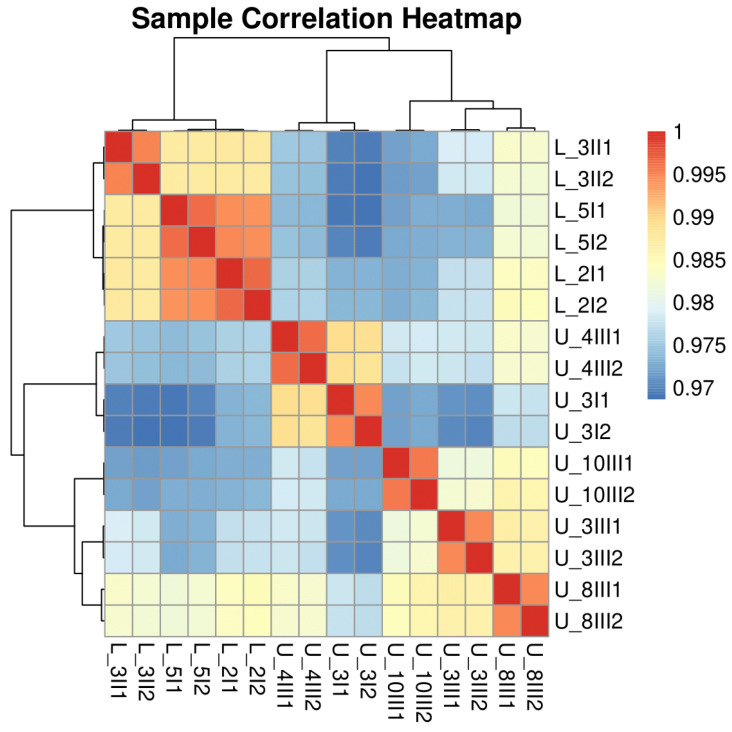
Sample correlation heatmap depicting pairwise Pearson correlation coefficients between RNA-seq samples based on FPKM-normalized gene expression profiles. Technical replicates are indicated by 1 or 2 in the sample name; biological replicates are indicated by U or L in the sample name. Each square represents the correlation of a specific pair of samples, with the color scale ranging from blue (lower correlation, 0.97) to red (higher correlation, 1.0).

**Figure 2:**
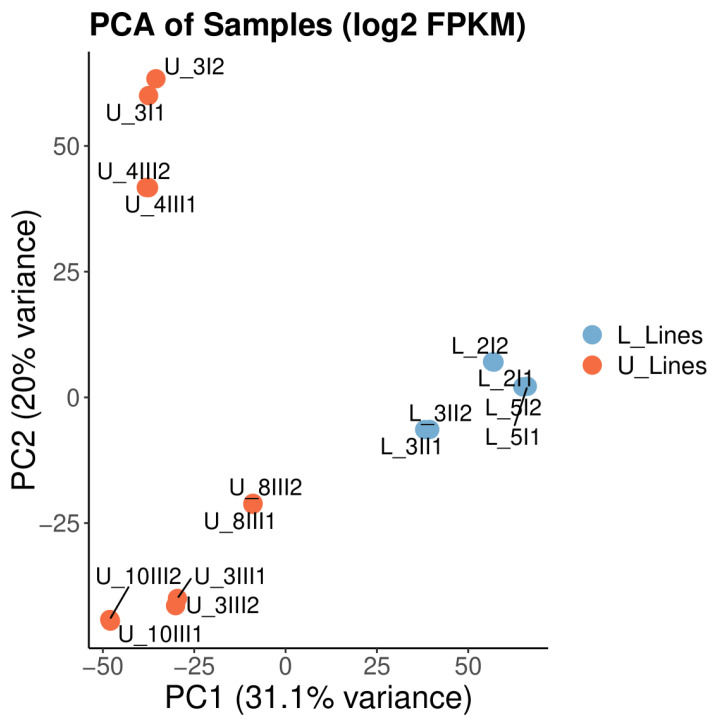
Principal component analysis (PCA) plot illustrating sample distribution based on log2-transformed FPKM gene expression profiles. Each point represents an individual sample, colored by biological replication group (blue for L_lines, orange for U_lines).

The data quality summary table obtained from Novogene shows a Q20 (%) score ≥97.70 for all samples and a total mapping rate ≥96.64%. A detailed data quality table for each sample is in the Supplementary Files (Supplementary Table S2). A comprehensive correlation analysis was performed among RNA-seq samples using FPKM-normalized gene expression data. The resulting heatmap and hierarchical clustering reveal that biological replicates cluster together and exhibit high pairwise correlation coefficients (≥0.97) (Figure 1), indicating high reproducibility and consistency across sequencing runs. Minor differences observed between distinct sample groups may reflect true biological variation. PCA of log2 FPKM values demonstrates robust separation between the two experimental groups (L_Lines and U_Lines), as depicted by the distinct clustering of samples on PC1 (Figure 2). Replicates within each group cluster closely, underscoring the consistency and reproducibility of gene expression profiles within experimental conditions. The substantial variance captured by PC1 and PC2 suggests that group identity is a key driver of gene expression variation, supporting a strong biological effect and validating the experiment’s design. Overall, the data quality is robust, supporting downstream comparative and functional analyses.

### 3.2. Differentially Expressed Genes

Differential expression analysis of a total of 6462 genes compared shows 2837 significant genes (1476 upregulated and 1361 downregulated) with adjusted *p*-values below 0.05 (the histogram of raw *p*-value distribution of all genes is in Supplementary Figure S1). Lists of differentially expressed genes (DEGs) are provided in Supplementary Materials (Supplementary Table S3). In the comparison of expressions of U and L lines, positive FC denotes higher expression in U_lines. Among the significantly expressed genes (FDR < 0.05), 1050 show large effect sizes (|log_2_FC| > 0.5), with 698 upregulated in U lines and 352 in L lines. All DEGs are listed in Supplementary File, Table S3.

**Figure 3:**
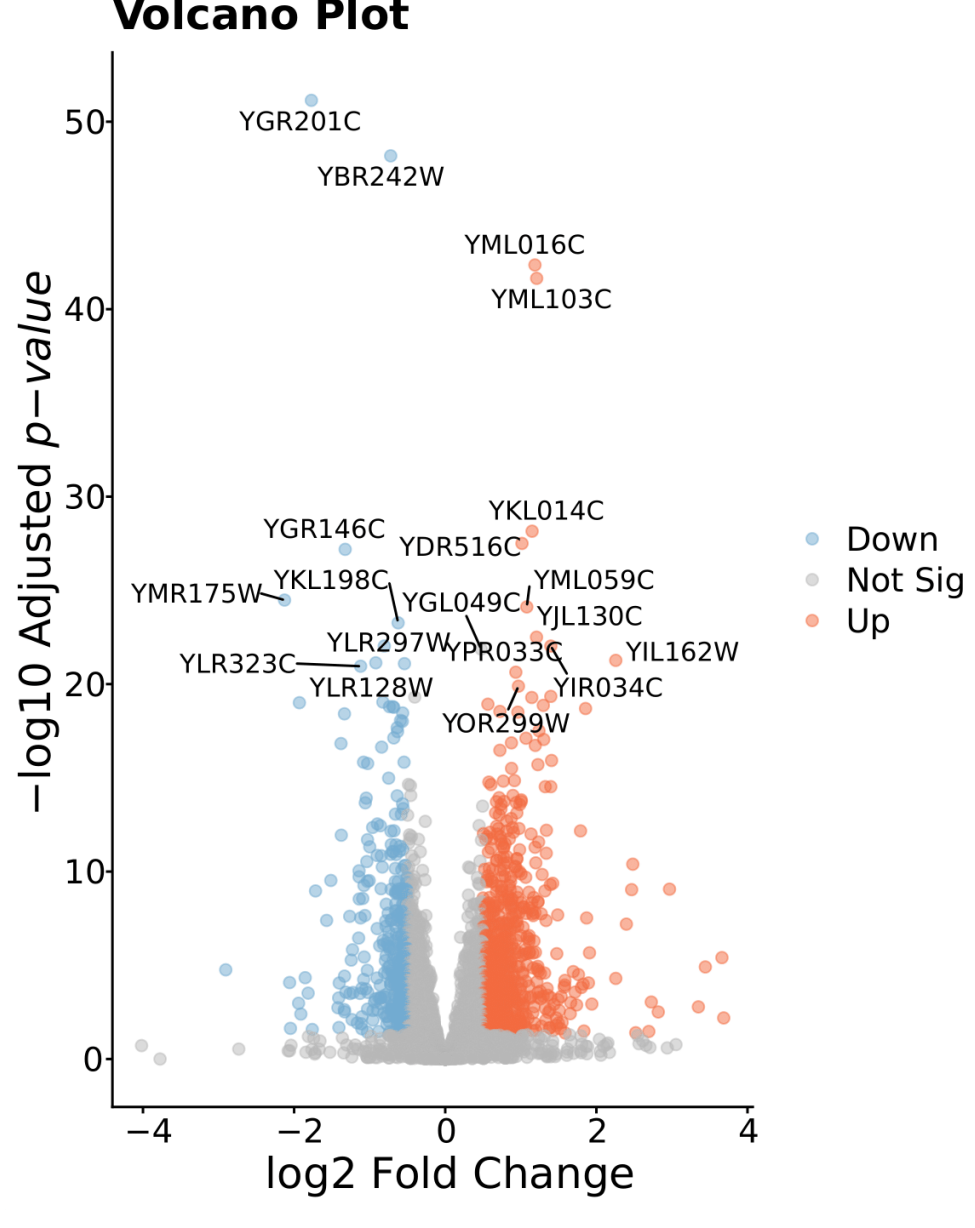
Volcano plot comparing gene expression between U_line and L_line groups of clones. Colored dots represent 1050 genes with increased expression in L (orange—698 genes) and U (blue—352 genes) groups (FDR < 0.05 and |log_2_FC| ≥ 0.5). Gray dots indicate a non-significant gene (YGL049C). X-axis (log_2_ fold--change): positive values indicate genes upregulated in U_lines versus L_lines; negative values indicate downregulation in U_lines versus L_lines. A total of 20 genes with the highest significance are annotated by name.

Focusing specifically on genes that exceed both thresholds (|log_2_ FC| ≥ 1 and −log_10_ *p*-value ≥ 20, corresponding roughly to *p* ≤ 1 × 10^−20^), there are nine genes most significantly downregulated in U_lines versus L_lines (Figure 3). Downregulation of these genes suggests a coordinated suppression of nucleotide metabolism, stress response factors, and protein modification machinery. YMR175W (SIP18) is a phospholipid-binding hydrophilin essential for osmotic stress tolerance, YKL198C (PTK1) is a putative serine/threonine kinase that regulates polyamine (spermine) uptake, YLR128W (DCN1) is a scaffold-type E3 ligase component required for cullin neddylation, YOR352W (TFB6) is a nonessential TFIIH subunit that facilitates dissociation of the Ssl2 helicase during transcription initiation, and YLR323C (CWC24) is a general splicing factor required for stable U2 snRNP binding to primary transcripts. Three ORFs (YGR201C, YLR297W, and YLR297W) encode uncharacterized proteins of unknown function; YBR242W is a 5′-deoxynucleotidase involved in deoxyribonucleoside monophosphate degradation, while YGR146C (ECL1) is a gene whose overexpression is known to extend chronological lifespan [27].

The eleven genes most significantly upregulated in U versus L lines of cells encompass diverse functions critical for metabolic maintenance, stress resistance, and cellular integrity. YML016C (PPZ1), a Ser/Thr phosphatase, regulates potassium homeostasis, pH balance, and cell cycle progression under stress. YKL014C (URB1) is essential for 60S ribosomal RNA processing and large-subunit biogenesis. YJL130C (URA2) and YIR034C (LYS1) sustain de novo pyrimidine and lysine biosynthesis, respectively, supporting nucleotide and amino acid pools during nutrient limitation. YML059C (*NTE1*) mediates phosphatidylcholine turnover, modulating Opi1p localization to repress phospholipid biosynthesis. YIL162W (*SUC2*) enables extracellular sucrose hydrolysis for alternative carbon uptake. YOR299W (BUD7) participates in exomer-dependent cargo sorting and bud-site selection, reinforcing cell wall assembly in the stationary phase. YPR033C (*HTS1*) encodes the histidyl-tRNA synthetase responsible for charging tRNA^His in both mitochondria and cytoplasm. YDR516C (EMI2), although sporulation-specific, is upregulated in U-enriched lines, suggesting a potential link between meiotic factors and quiescence. Finally, YML103C (NUP188) forms part of the nuclear pore complex scaffold, modulating nucleocytoplasmic transport and nuclear integrity during G_0_. Together, these upregulated genes define a program that integrates ion and pH homeostasis, macromolecular synthesis, membrane remodeling, and stress-adaptive structures to optimize survival and frequent exit from quiescence.

### 3.3. Ontology of the RNA-Seq Upregulated in L_ or U_line Genes Show Differences Between Quiescence Cells from Populations of Different Enrichment Histories

**Figure 4:**
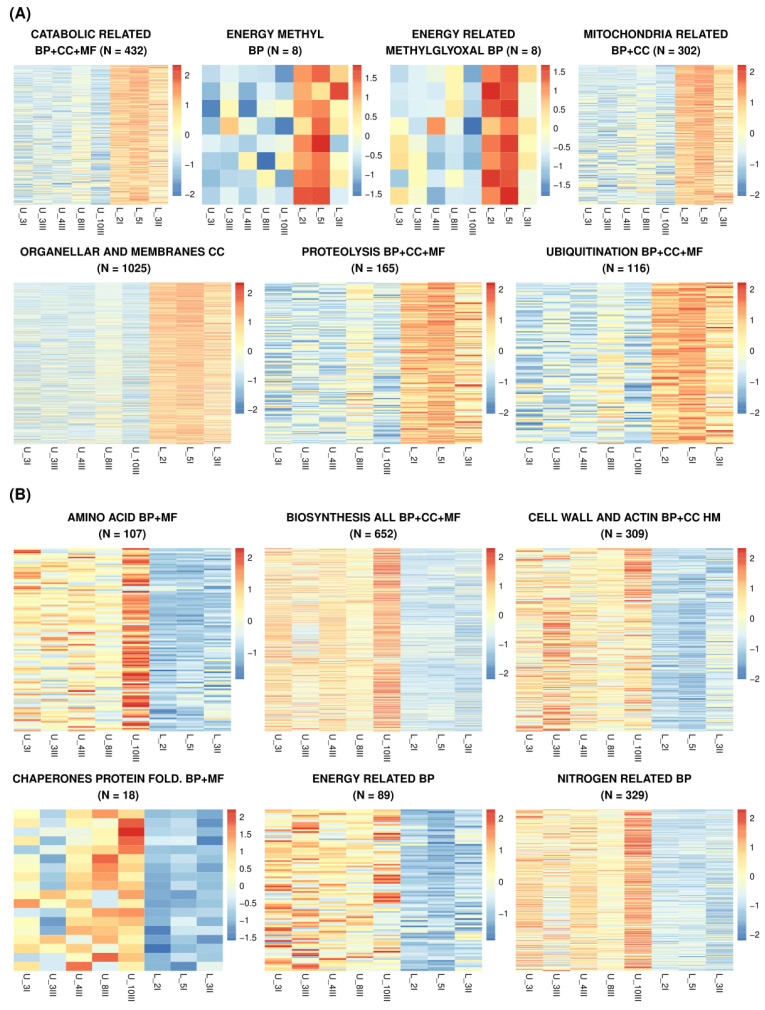
Heat maps of the GO categories of differentially expressed genes in groups of clones from U and L enriched lines. (**A**) downregulated in U (upregulated in L) (**B**) upregulated in U (downregulated in L). Expression values (FPKM) were scaled per gene (row z-score). The color scale represents z-scores. Number of genes per panel was indicated in the title.

Sets of all genes with different expression for groups of clones (U_lines and L_lines) were compared with background lists of all ORFs from the RNA-seq analysis. Following GOrilla analysis, the groups with increased transcribed genes (of FDR less than 0.01) were grouped within several GO categories (process, component, and function) (data are provided in Supplementary Table S4. Heat_Map_RNAseq_GO). Six groups of Gene Ontology terms are upregulated in the U-line group of clones and encompass all three categories: biological process (BP), molecular function (MF), and cellular component (CC). These categories are as follows: amino acid-related (BP and MF), biosynthesis (translation, biosynthesis, RNA processing, and ribosomes) (BP, MF, and CC), cell wall and actin filaments (BP and CC), chaperones (BP and MF), energy-related processes, and nitrogen-related processes (Figure 4A).

A total of seven groups were identified as being overexpressed within the L_line group of clones. These groups are as follows: catabolic-related (BP, MF, and CC), CoA-related (BP), methylglyoxal (BP), mitochondria-related (BP and CC), organellar- and membrane-connected (CC), and proteolysis and ubiquitination (BP, MF, and CC) (Figure 4B).

## 4. Discussion

In the last decade, major progress has been made to increase understanding of yeast quiescence entry, maintenance, and exit, as described in comprehensive reviews [13,28,29]. However, there are still many important questions left: *Is quiescence a single state, or could there be many different types of quiescence? How different is quiescence when it is triggered by different signals? What is the degree of cell-to-cell variation in signaling, chromatin dynamics, and gene expression during quiescence entry, maintenance, and exit?*

Here, a comparison is made between the transcriptomes of the lower fraction (L) quiescent cells within the population that experienced different environmental stress conditions. It was determined that the transcriptome reflects the ~300 generations of evolutionary history of the population, thereby rendering it more prepared for environmental conditions to which it has been previously exposed. The downregulation of genes observed in quiescent L cells from U _lines reflects a history of elimination of all lower fraction QL cells before regrowth. This has resulted in an elevated prevalence of U types of Q cells in comparison to their ancestral counterparts, as well as L-line enrichment.

A total of seven GO groups were identified as being downregulated in U_line groups (Figure 4). A subset of the downregulated genes within these categories appears to be of possible significance. The downregulation of *DCN1*, a crucial component in the orchestration of cullin neddylation, emerges as a salient feature. A reduction in *DCN1* expression has been shown to attenuate ubiquitin–proteasome pathway activity. *DCN1* is one of the 116 genes involved in the ubiquitination process (PP, CM, and MF, as shown in Figure 4), thereby decelerating protein turnover and preserving ATP. This energy-saving strategy has also been observed in quiescent mammalian cells, where proteostasis adjustments are implicated in supporting long-term survival [30,31] which might explain why it is more expressed in the L_lines, which are prepared for longer quiescence periods [18].

Concurrent repression of *TFB6*, which encodes a previously unidentified, dissociable subunit of the yeast general transcription factor TFIIH complex, might be meaningful. The TFIIH subunit is critical for controlling the release of the Ssl2 helicase during transcription initiation [32], suggesting an active remodeling of the transcriptional machinery. By shifting TFIIH away from its holoenzyme form, cells more effectively enforce the global transcriptional repression characteristic of G_0_ entry and maintenance. Although Tfb6 itself is nonessential under standard growth conditions, its function may hypothetically be analogous to other nonessential regulators (e.g., Xbp1 and Msa1/2) that fine-tune the chromatin and transcriptional reprogramming underlying quiescence [33].

Loss of Cwc24p following decreased *CWC24* expression might mirror the general downshift in spliceosomal components noted in quiescent yeast, reducing energy-intensive mRNA maturation processes when transcription of intron-containing genes nearly ceases. Such coordinated repression of splicing factors has been proposed to prevent the accumulation of unprocessed transcripts and to reallocate resources to stress resistance [34]. Stress response pathways are also modulated as *SIP18* and *PTK1* encode proteins essential for osmotic stress tolerance and polyamine uptake, respectively. Their downregulation suggests that quiescent cells deprioritize adaptation to external environmental fluctuations in favor of basal maintenance, reflecting a narrowed physiological focus [19,35].

In the course of quiescence research, uncharacterized genes and open reading frames (ORFs) have been frequently identified. This phenomenon was also observed in our study, particularly with YGR201C, YLR297W, and *ECL1*, likely representing quiescence-specific regulators. It is worth noting that *ECL1* overexpression extends chronological lifespan, hinting that its downregulation in U_lines might fine-tune survival pathways or sensitize cells to reactivation cues [27].

We identified six groups of Gene Ontology terms that were upregulated in the U-line group of clones (Figure 2). In the U-line enrichment set, a few upregulated genes seem to have a role in quiescence. During quiescence, cells must maintain membrane integrity over extended periods while adapting to changing metabolic conditions. Proper lipid homeostasis is crucial for membrane stability, and the cellular adaptations required for long-term survival are conserved from bacteria to humans [36]. Thus, *NUP188*, involved in membrane lipid homeostasis upregulation, might represent a strategic reprogramming of lipid metabolism to stabilize membranes and suppress unnecessary biosynthesis and RNA processing. It may also prepare for efficient exit from G_0_, which is expected to happen more often for U_line cells.

Another potentially interesting gene is *SUC2*, which encodes the secreted enzyme invertase, which hydrolyzes extracellular sucrose into glucose and fructose. Its transcription is tightly repressed in high-glucose conditions by the Mig1 repressor and derepressed when glucose levels fall via activation of the Snf1 kinase pathway and associated factors (e.g., Cat8 and Sip4), which also regulate gluconeogenic and stress-protective genes during entry into quiescence. Thus, SUC2 induction reflects broader remodeling of transcriptional networks that balance energy conservation, stress tolerance, and readiness for reactivation. By maintaining a low-flux steady-state of sugar uptake, invertase activity may keep key signaling pathways—such as Ras/PKA or TORC1—at threshold levels that facilitate rapid sensing of improved nutrient conditions, shortening the lag phase upon return to growth [37]. *SUC2* upregulation in U_lines might represent a strategic adaptation: it enables scavenging of alternative carbon sources, feeds storage polysaccharide synthesis, integrates with global stress and nutrient-sensing networks, and primes cells for swift reentry into the cell cycle when nutrients become available.

During quiescence, maintaining proper ion homeostasis becomes critical for long-term survival and stress tolerance. *PPZ1* encodes a serine/threonine phosphatase that regulates K+ transport and pH homeostasis. Research demonstrates that Ppz1 controls the activity of Trk K+ transporters, affecting membrane potential, intracellular pH, and cell cycle progression. In addition, overexpression of *PPZ1* causes a pronounced slow-growth defect [38]), which might be positive in U enriched lines.

While overall ribosome biogenesis decreases during quiescence, specific rRNA processing pathways may be maintained or even enhanced to support the production of specialized ribosomes adapted for the quiescent state [39]. This might explain the upregulation of *URB1*, which is involved in ribosomal RNA processing. Such regulation allows for the tailored production of ribosomes capable of handling the unique cellular environment and requirements of quiescent cells [40]. The de novo pyrimidine pathway is regulated at the enzymatic level by feedback inhibition of the initial enzyme, Ura2, via the final product, UTP.

Additionally, several pathway genes are upregulated at the transcriptional level in response to pyrimidine starvation. *URA2* encodes carbamoylphosphate synthetase for pyrimidine biosynthesis, and *LYS1* encodes saccharopine dehydrogenase for lysine biosynthesis. The upregulation of these biosynthetic enzymes during quiescence suggests that, despite overall metabolic downregulation, cells maintain the capacity to produce essential building blocks, ensuring readiness for cell cycle reentry when nutrients become available [41].

*SED1* encodes a major structural cell wall protein specifically expressed in the stationary phase. Research demonstrates that Sed1p becomes the most abundant cell wall protein in stationary-phase cells, accounting for 30% of extractable cell wall proteins. *SED1* expression increases 2.7-fold in the stationary phase and is required for lytic enzyme resistance. This protein exemplifies the specialized cell wall adaptations necessary for quiescent cell survival [6,42]. While specific mechanistic details for *TBS1*—General Stress Tolerance are limited, its identification as enhancing general stress tolerance mechanisms aligns with the comprehensive stress resistance programs activated during quiescence entry.

## 5. Conclusions

Our study confirms the assumption that quiescence in *Saccharomyces cerevisiae* is not a uniform physiological state but rather a dynamic condition shaped by present and past conditions. Our comparative transcriptomic analysis of dense (L) quiescent cells from populations with distinct enrichment histories reveals several genes that show differential expression patterns reflecting approximately 300 generations of selection pressure. The observed transcriptional differences encompass critical cellular processes, including energy metabolism, stress response, protein homeostasis, and cell wall maintenance, indicating that quiescent cells retain remarkable adaptive plasticity even within morphologically homogeneous fractions.

The contrasting gene expression profiles between L cells within U_ and L_ lines support a model of quiescence as an evolvable trait rather than a fixed cellular program. U_lines upregulated genes associated with biosynthesis, translation machinery, and rapid reactivation pathways, reflecting adaptation to environments favoring quick exit from quiescence. Conversely, L_lines showed enhanced expression of catabolic processes, mitochondrial functions, and proteolysis machinery, consistent with optimization for prolonged survival during extended starvation. These findings challenge the notion of a universal “core quiescent program” and instead reveal that quiescent cells can fine-tune their molecular machinery based on ecological context and selection history. Understanding this transcriptional plasticity has important implications for microbial ecology, biotechnology applications, and our broader comprehension of cellular dormancy mechanisms across diverse biological systems.

## Data Availability

Transcriptomic data were deposited in GEO (Gene Expression Omnibus) Series GSE309454.

## Supplementary Materials

The following supporting information can be downloaded at https://www.mdpi.com/article/10.3390/biom15111588/s1. Table S1. Single-nucleotide variation present in the clones used in this study. Table S2. Data quality summary table obtained from Novogene. Figure S1. Histogram of the raw *p*-value distribution of all genes. Table S3. Lists of differentially expressed genes (DEGs). Table S4. Heat_Map_RNAseq_GO.
