# Supplementary material for: Ecological History Shapes Transcriptome Variation in Quiescent Saccharomyces cerevisiae
Source: Biomolecules. 2025 Nov 12;15(11):1588. doi: 10.3390/biom15111588 (PMC12650442; doi:10.3390/biom15111588)
Supplement: Supplementary file 1 [file biomolecules-15-01588-s001.zip › biomolecules-3922832-supplementary/Suplementary_Files_Biomolecules/Table S2.pdf]

Sup. Table S2. Data quality summary table obtained from Novogene

Sample name	Raw reads	Clean reads	Raw bases	Clean bases	Error rate(%)	Q20(%)	Q30(%)	GC content(%)
U_3I1	22577580	22190173	6.8G	6.7G	0.03	98.02	94.12	41.03
U_3I2	23657401	23309291	7.1G	7.0G	0.03	98.00	94.06	40.99
L_2I1	22976189	22612170	6.9G	6.8G	0.03	97.91	93.91	41.13
L_2I2	22348655	22047786	6.7G	6.6G	0.02	98.18	94.47	41.08
L_5I1	21859680	21492266	6.6G	6.4G	0.03	98.01	94.14	41.20
L_5I2	23994932	23604396	7.2G	7.1G	0.03	97.86	93.79	41.18
L_3II1	22991855	22550250	6.9G	6.8G	0.03	97.98	94.10	40.99
L_3II2	20733624	20383091	6.2G	6.1G	0.02	98.03	94.21	41.02
U_3III1	23742721	23358238	7.1G	7.0G	0.03	97.82	93.67	41.05
U_3III2	22242942	21888057	6.7G	6.6G	0.02	98.18	94.55	41.06
U_4III1	25824239	25442160	7.7G	7.6G	0.03	97.98	94.06	41.02
U_4III2	20379615	20108105	6.1G	6.0G	0.03	97.98	94.08	41.10
U_8III1	22670305	22401064	6.8G	6.7G	0.02	98.09	94.26	41.14
U_8III2	21382707	20957280	6.4G	6.3G	0.03	97.93	93.97	41.21
U_10III1	20608361	20256133	6.2G	6.1G	0.03	97.73	93.48	41.12
U_10III2	22869532	22486735	6.9G	6.7G	0.02	98.01	94.16	41.14

(1) Sample name: sampleID. Last digit indicates technical repetition.

(2) Raw reads: reads count from the raw data, four rows as a unit, with statistics of reads count for every sequencing.

(3) Clean reads: Clean data are reads count filtered from raw data. Statistics method is similar with raw reads. All the following analysis is based on clean data.

(4) Raw bases: Base number of raw data. (number of raw reads) * (sequence length), converting unit to G.

(5) Clean bases: Base number of raw data after filtering. (number of clean reads) * (sequence length), converting unit to G.

(6) Error rate(%): base error rate of whole sequencing.

(7) Q20(%): The percentage of the bases whose Q Phred values is greater than 20. (Number of bases with Q Phred value > 20) / (Number of total bases) *100.

(8) Q30(%): The percentage of the bases whose Q Phred values is greater than 30. (Number of bases with Q Phred value > 30) / (Number of total bases) *100.

(9) GC content(%): The percentage of G&C base numbers of total bases.(G&C base number) / (Total base number)*100.
