# Supplementary figures and images for: Ecological History Shapes Transcriptome Variation in Quiescent Saccharomyces cerevisiae
Source: Biomolecules. 2025 Nov 12;15(11):1588. doi: 10.3390/biom15111588 (PMC12650442; doi:10.3390/biom15111588)

Histogram of raw p-value distribution of all genes

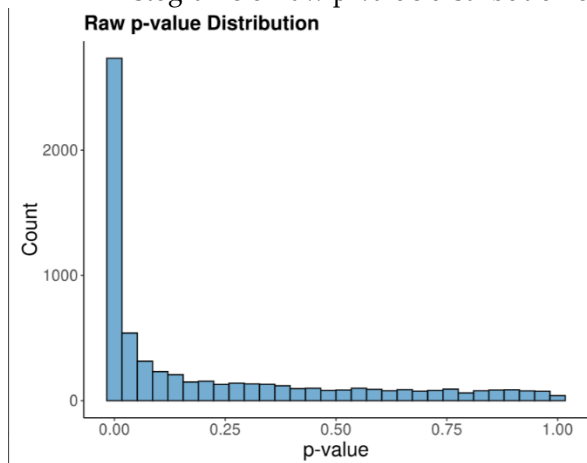

Supplement: Supplementary file 1 [file biomolecules-15-01588-s001.zip › biomolecules-3922832-supplementary/Suplementary_Files_Biomolecules/Figure S1.pdf]
